# Biochemical mechanisms preventing wilting under grafting: a case study on pumpkin rootstock grafting to wax gourd

**DOI:** 10.3389/fpls.2024.1331698

**Published:** 2024-05-02

**Authors:** Houlong Fu, Junyu Fu, Bin Zhou, Haolong Wu, Daolong Liao, Zifan Liu

**Affiliations:** ^1^School of Tropical Agriculture and Forestry, Hainan University, Haikou, China; ^2^Tropical Crops Genetic Resources Institute, Chinese Academy of Tropical Agricultural Sciences, Haikou, China; ^3^Institute of Vegetables, Hainan Academy of Agricultural Sciences, Haikou, China

**Keywords:** wax gourd, *Fusarium oxysporum*, grafting, pathogenic fungi, plant enzymes, secondary metabolites

## Abstract

Wax gourd wilt is a devastating fungal disease caused by a specialized form of *Fusarium oxysporum* Schl. f. sp. *benincasae* (FOB), which severely restricts the development of the wax gourd industry. Resistant rootstock pumpkin grafting is often used to prevent and control wax gourd wilt. The “Haizhan 1” pumpkin has the characteristic of high resistance to wilt, but the mechanism through which grafted pumpkin rootstock plants acquire resistance to wax gourd wilt is still poorly understood. In this study, grafted wax gourd (GW) and self-grafted wax gourd (SW) were cultured at three concentrations [2.8 × 10^6^ Colony Forming Units (CFU)·g^−1^, 8.0 × 10^5^ CFU·g^−1^, and 4.0 × 10^5^ CFU·g^−1^, expressed by H, M, and L]. Three culture times (6 dpi, 10 dpi, and 13 dpi) were used to observe the incidence of wilt disease in the wax gourd and the number of *F. oxysporum* spores in different parts of the soil and plants. Moreover, the physiological indices of the roots of plants at 5 dpi, 9 dpi, and 12 dpi in soil supplemented with M (8.0 × 10^5^ CFU·g^−1^) were determined. No wilt symptoms in GW. Wilt symptoms in SW were exacerbated by the amount of FOB in the inoculated soil and culture time. At any culture time, the amount of FOB in the GW soil under the three treatments was greater than that in the roots. However, for the SW treatments, at 10 dpi and 13 dpi, the amount of FOB in the soil was lower than that in the roots. The total phenol (TP) and lignin (LIG) contents and polyphenol oxidase (PPO) and chitinase (CHI) activities were significantly increased in the GW_M_ roots. The activities of phenylalanine ammonia lyase (PAL) and peroxidase (POD) initially decreased but then increased in the GW_M_ roots. When the TP content decreased significantly, the LIG content and PAL and CHI activities increased initially but then decreased, whereas the PPO and POD activities did not change significantly in the SW_M_ roots. The results indicated that the roots of the “Haizhan 1” pumpkin stock plants initiated a self-defense response after being infected with FOB, and the activities of PPO, POD, PAL, and CHI increased, and additional LIG and TP accumulated, which could effectively prevent FOB infection.

## 1 Introduction

Wax gourd (*Benincasa hispida* (Thunb) Cogn.) is an annual vine of the Cucurbitaceae family with high nutritional and medicinal value (Islam et al., 2021; Yang et al., 2023). It is native to South China and East India and is now widely cultivated in subtropical and tropical Asia. The planting area of wax gourds in China exceeds 330,000 hm^2^ (Xie et al., 2020). Due to the popularity of wax gourd planting in China in recent years, the planting area in various regions has expanded, especially in Guangxi and Hainan, which are the major planting areas for wax gourds in China. With the increase in multiple planting indices and the limitation of wax gourd varieties, the occurrence of wax gourd disease is becoming increasingly prominent and frequent, especially wax gourd wilt, which has become the first fatal disease to wax gourd (He et al., 2021). Wax gourd wilt has a severe effect on the wax gourd industry, for which a 20%–30% reduction in production would occur with a mild disease, and in severe conditions, it will lead to a more than 60% reduction in production (Shi et al., 2022). Wax gourd wilt has become an important factor that limits the healthy development of the wax gourd industry.

Wax gourd wilt is a devastating soil-borne fungal disease caused by a specialized form of *Fusarium oxysporum* Schl. f. sp. *benincasae* (Wang and Fang, 2021). Under suitable conditions, the conidium enters the plant through the root wound or from the tip cells of the root hair, obstructing the transportation of water and nutrients. Generally, the whole plant wilts and dies within 3–5 days after infection (Wang, 2018). It is almost impossible to eradicate the pathogen once it successfully propagates in the soil (Dita et al., 2018). Currently, the most widely used methods for controlling soil-borne diseases are the use of chemical fungicides and cultivation of resistant plant varieties. Chemical control measures not only require huge manpower and material resources, but also pollute the environment; thus, satisfactory results cannot be achieved. For disease-resistant plant cultivation, it takes a long time to select and breed a new variety of wax gourds that are resistant to wilt diseases. Therefore, there is an urgent need to find a green and efficient method to control wax gourd wilt.

Grafting is an agricultural technology that not only retains the good traits of scion varieties, but also uses the unbeneficial characteristics of the rootstock to overcome soil-borne diseases caused by crops. Disease resistance of grafted plants is closely correlated with the selection of resistant root stocks. For example, Li (2022) found that black-seeded pumpkin is resistant to wax gourd wilt and can be used as a grafted rootstock to effectively control wilt. Zeng et al. (2004) studied the effect of black-seed pumpkin as a rootstock on cucumber wilt resistance and reported that, compared with cucumber rootstock, black-seed pumpkin rootstock can significantly promote cucumber growth and fruit growth and improve nutrient absorption efficiency, thus preventing wilt. Therefore, rootstock pumpkin grafts are an important measure for preventing and controlling the occurrence of wax gourds.

**Figure 1:**
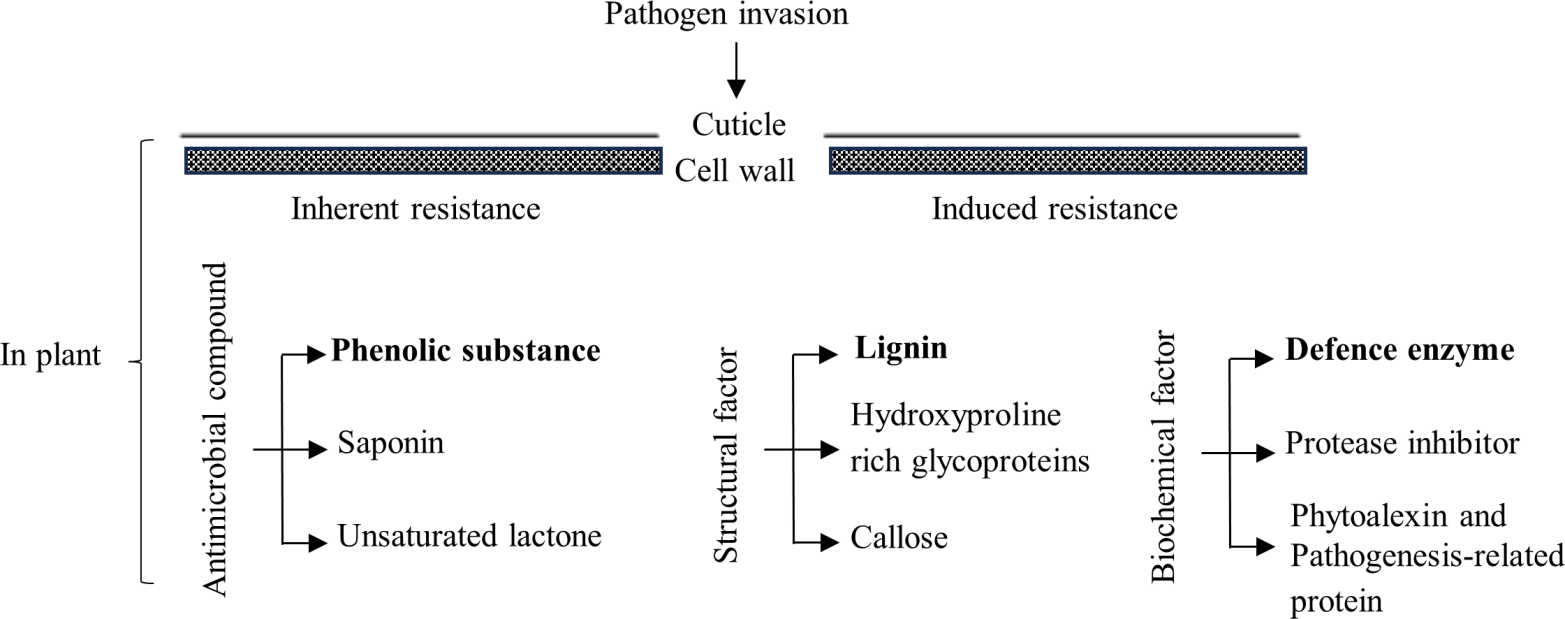
Mechanisms of plant disease resistance.

When plants are infected by pathogenic bacteria or fungi, corresponding changes are triggered by antimicrobial compounds, inherent structural barriers, multiple defense enzymes, and disease resistance-related genes, which rely on their inherent immune system and induce resistance to obtain systemic resistance (**Figure 1**). For example, certain compounds in plants inhibit the growth of fungi, and most phenolic substances promote or inhibit fungi. The invasion of fungal pathogens into crops, such as cotton, tomatoes, bananas, and potatoes, results in the rapid oxidation of phenolic substances in crop cells, with consequent lignification and suberization of the cells and cell death (Beckman, 2000). The cell wall plays a fundamental role in the structural barrier inherent to plants against pathogens. Lignin is one of the components of plant cell wall. Invasion by pathogens stimulates lignin synthesis, which in turn thickens the cell wall and increases the plant’s resistance to disease (Siddaiah et al., 2017). The defense enzymes include superoxide dismutase (SOD), catalase (CAT), peroxidase (POD), polyphenol oxidase (PPO), phenylalanine ammonia lyase (PAL), and chitinase (CHI) (Tian et al., 2015; Guo et al., 2018). For example, the activities of PPO, PAL, and POD in banana (Hu, 2006), PPO, PAL, CAT, CHI, POD, and SOD in bitter gourd (Zhao et al., 2013; Li et al., 2019b), and SOD, POD, PPO, PAL, and CAT in watermelon (Chen and Chen, 2019) were all positively correlated with resistance to *Fusarium* wilt infestation. Zeng (2021) reported that metabolic pathways such as phenylpropanoid synthesis and phenylalanine metabolism were involved in the anti-wilt reaction of wax gourd used for rootstock. Our research group found that the “Haizhan 1” pumpkin had high resistance to wilt, and the wilt disease index of grafted wax gourds was 0 in a field test (Yuan et al., 2021). However, the mechanism by which grafted plants of the “Haizhan 1” pumpkin rootstock acquire resistance to wax gourd wilt is still poorly understood. The prerequisite for invasion, colonization, and expansion of FOB is infection of the root system. To provide a theoretical basis for germplasm optimization and cultivation control of wax gourds, the incidence and FOB quantity distribution of grafted and self-grafted wax gourds were observed and measured by simulating soils with different concentrations of FOB. Physiological indices of root disease resistance in grafted wax gourds were measured in soil supplemented with medium concentrations of FOB.

## 2 Materials and methods

### 2.1 Materials

The *F. oxysporum* Schl. f. sp. *benincasae* physiological subspecies No. 1 (expressed by FOB) was obtained from the Vegetable Research Institute of the Guangxi Academy of Agricultural Sciences.

The test soil was extracted from Luofu village, Ruixi town, Chengmai County (19°74’90.29’’N–110°10’55.41’’E) and had the following basic and chemical properties: pH 5.94, 0.51% organic matter, 28.1 mg·kg^−1^ alkaline hydrolysis nitrogen, 103.3 mg·kg^−1^ quick-acting phosphorus, and 129.6 mg·kg^−1^ quick-acting potassium. The test soil debris was removed, and the plants were screened through an 80 mesh and sterilized three times for 2 h, after which the sterilized soil was obtained after air drying (Wang et al., 2013).

The wax gourd variety “Tiezhu 2” was obtained from the Vegetable Research Institute of the Guangdong Academy of Agricultural Sciences. The pumpkin variety “Haizhan 1” was provided by the Vegetable Research Institute the of Hainan Academy of Agricultural Sciences. The seedlings were obtained from the Chengmai Base of the Hainan Academy of Agricultural Sciences. The seeds of the wax gourd and pumpkin rootstocks were soaked for 12 h on the first day (24 November) and after 5 days (28 November 2022), respectively, after which the seeds were visibly germinated and subsequently planted in a tray with 60 holes. After 4 days (27 November), the seeds of the wax gourd used as scions were soaked for 12 h and then sown in trays after visible germination. Grafted Wax gourd (GW) and Self-Grafted Wax gourd (SW) were obtained using the grafting method when the scions grow to two flat cotyledons, and the first true leaf of the rootstock forms a fusiform shape (Sabry et al., 2022). When the plants had grown two leaves, they were moved to the Cultivation System Research Laboratory of the Agricultural Science Building, Hainan University for seedling experiments on 13 December 2022.

### 2.2 Preparation of spore suspension

Potato dextrose agar (PDA) and potato dextrose broth (PDB) were prepared for culturing FOB (Attia et al., 2020; Westphal et al., 2021). After culturing FOB in PDA medium for 7 days, the mycelia of FOB were selected and transferred to PDB media. The inoculated PDB culture medium was placed in a constant-temperature oscillator for 3 days (25 °C, rotating speed 150 r·min^−1^). The spore suspension was obtained by filtering absorbent cotton. The concentration of spores was measured using a microscope and hemocytometer, and the final concentration was 2.75 × 10^7^ CFU·g^−1^. The spore suspension was diluted to ratios of 1:4 and 1:16, and the spore suspension diluent was set aside.

### 2.3 Preparation of inoculated soils

The spore suspension and its diluent were poured into sterilized soil at a ratio of 6 g:1 ml, mixed well, and inoculated soil of three concentrations was obtained. The concentrations of *Fusarium* wilt fungus in the three kinds of soil obtained by the plate counting method were 2.8 × 10^6^ CFU·g^−1^, 8.0 × 10^5^ CFU·g^−1^, and 4.0 × 10^5^ CFU·g^−1^ (represented by H(igh), M(edium), and L(ow), respectively). The inoculated soil samples with different concentrations of FOB were subsequently transferred to plastic pots (120 g per container).

### 2.4 Experimental design

In the experiment, Grafted Wax gourds (GW) and self-grafted Wax gourds (SW) were grown in soil supplemented with three concentrations of FOB (2.8 × 10^6^ CFU·g^−1^, represented by H; 8.0 × 10^5^ CFU·g^−1^, represented by M; and 4.0 × 10^5^ CFU·g^−1^, represented by L), which were represented by GW_H_, GW_M_, GW_L_, SW_H_, SW_M_, and SW_L_ respectively. Seedlings of the same size were selected on 18 December 2022, and after the roots were cleaned, the plants were transplanted into a square nutrient pot (length × width × height = 6.5 cm × 4 cm × 6.5 cm) stuffed with FOB-infected soil. The experiment included 50 plants per treatment, with each plant planted in a single pot. The plants were cultured in a light incubator at 26 °C/21 °C (12 h/12 h) with a light intensity of 2,000 lx. No watering was required for 2 days before culture, and the water consumed by the plants was replenished every two days before they were fertilized.

### 2.5 Determination indices and methods

#### 2.5.1 Observation of plant disease

The growth and incidences of plants were observed and photographed daily.

##### 2.5.1.1 Determination of the number of F. oxysporum

The culture times were 6 dpi, 10 dpi, and 13 dpi for the specific dates of 24 December, 28 December, and 31 December 2022, respectively. The soil on the root surface was first rinsed and subsequently rinsed twice with sterile water. The roots, combined stems, and scions were clipped, and fresh weights were determined. Various parts of the weighed plant were cut and ground in a mortar. An appropriate amount of sterile water was added to replenish the mass to 10 g (plant mass + sterile water mass = 10 g), which was subsequently shaken for 30 min at a speed of 150 r·min^−1^, after which a 10^−1^ plant diluent was obtained. Similarly, all soil from the above three plants was poured into a dish and mixed, and 90 g of soil and 10 g of sterile water were weighed and sealed for 30 min at a rotating speed of 150 r·min^−1^, after which 10^−1^ soil suspensions were obtained. Diluents and soil suspensions from different parts of the plants with dilution gradients of 10^−2^, 10^−3^, and 10^−4^ were evenly coated on *F. oxysporum*-specific K2 media (Ou, 2019), cultured for 3 days, and counted. Each treatment was repeated four times. The number of *F. oxysporum* was calculated according to the following formula (Liu et al., 2021):


$$\text{Colony count}(\text{CFU}\cdot {\text{g}}^{-1})=\frac{\text{Colony average}\times \text{Dilution ratio}}{\text{Sample amount per dish}\times \text{Fresh sample}/\text{Dry soil quality}}$$

##### 2.5.1.2 Determination of the antimicrobial compound, physical barrier indicators and defense enzyme activities in roots

The culture times were 5 dpi, 9 dpi, and 12 dpi for the specific dates of 23 December, 27 December, and 30 December 2022, respectively. Plants treated with GW_M_ and SW_M_ (grafted wax gourd and self-grafted wax gourd soaked in medium concentration diluent) were randomly collected eight plants, and the roots of three plants were washed, placed them in kraft paper, dried at 80 °C to constant weight, ground and sieved through 30–50 meshes for the determination of total phenol (TP) (Wang et al., 2020) and lignin (LIG) (Liang et al., 2020). The roots of five plants were separately packed in frozen tubes (−80 °C) to determine physiological indices, such as polyphenol oxidase (PPO) (Li et al., 2019a), peroxidase (POD) (Dou et al., 2017), phenylalanine ammonia lyase (PAL) (Zhang et al., 2020), and chitinase (CHI) (Kadoo and Badere, 2017). These parameters were determined using a spectrophotometer (visible spectrophotometry) produced by Beijing Solarbio Technology Co., Ltd.

### 2.6 Data analysis

Before analysis, all raw data were summarized and classified using Microsoft Excel. Duncan’s new complex range method in DPS (9.05 edition) was used for multiple comparisons, and the number of *F. oxysporum* in different parts of the soil and plants, the secondary metabolic substances in the roots, and their defense enzyme activities were analyzed at a significance level of p<0.05. Correlation analysis in DPS (9.05 edition) was used to analyze the correlation between the secondary metabolites in roots and their defense enzyme activities, and the differences between the six different indices at p<0.05 and p<0.01. Finally, Origin 2023 was used for the mapping.

## 3 Results

### 3.1 Effects of pumpkin grafting on the symptoms of wax gourd wilt

**Figure 2:**
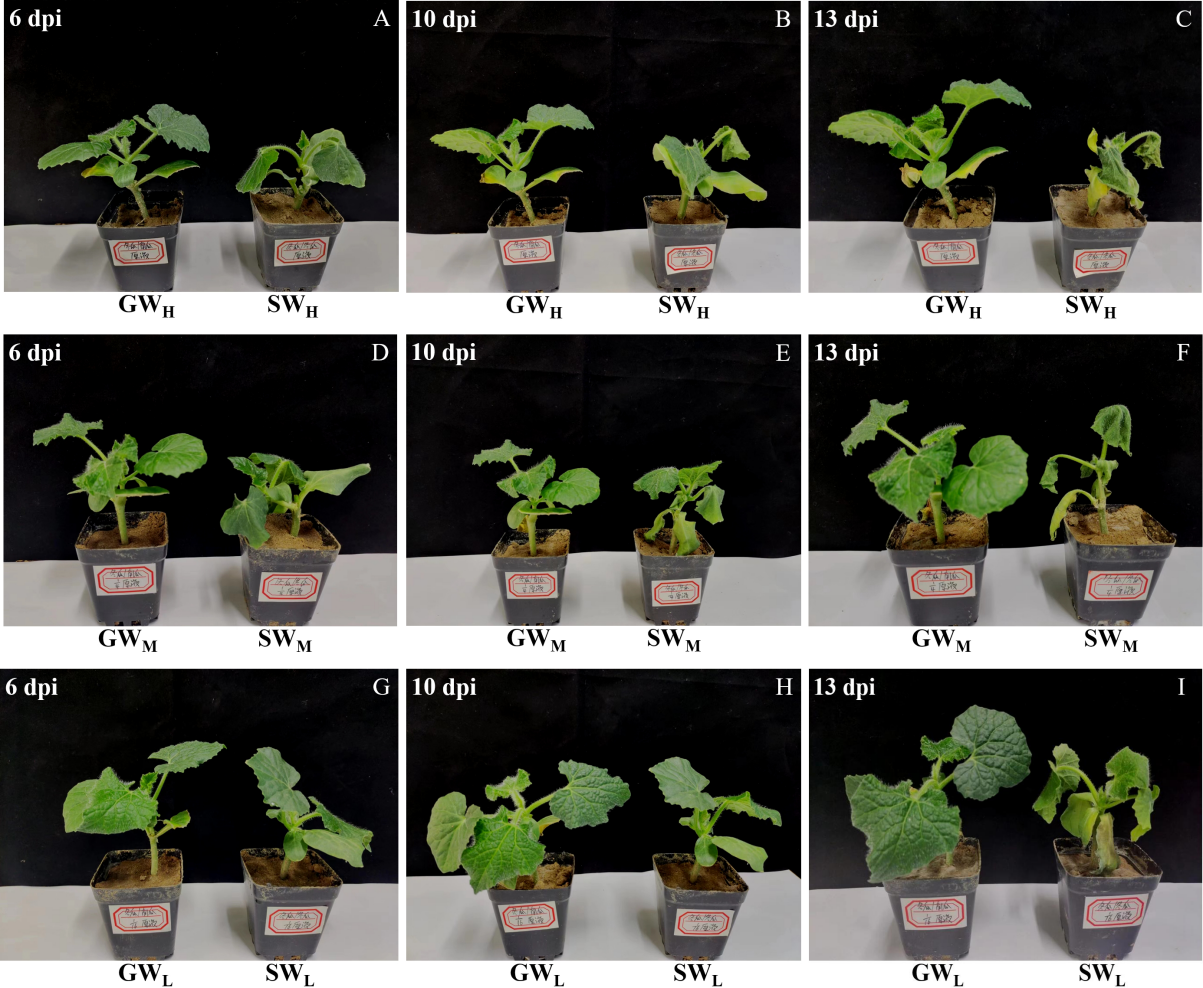
**(A–I)** Differences in the morphology of plant disease. dpi, days post inoculation, GW_H_, Grafted wax gourd high concentration, GW_M_, Grafted wax gourd medium concentration, GW_L_, Grafted Wax gourd low concentration, SW_H_, Self-grafted wax gourd high concentration, SW_M_, Self-grafted wax gourd medium concentration, SW_L_, Self-grafted wax gourd low concentration.

GW_H_, GW_M_, and GW_L_ seedlings did not exhibit symptoms of wilt disease at any time point. SW_H_ seedlings began to show disease symptoms at 6 dpi. SW_M_ seedlings began to show symptoms of the disease at 10 dpi and SW_L_ seedlings began to show symptoms of the disease at 13 dpi (**Figure 2**). In other words, the symptoms of wilt disease in SW plants worsened with increasing FOB numbers in the inoculated soil and prolonged cultivation time.

### 3.2 Effect of pumpkin grafting on the number of *F. oxysporum*

**Figure 3:**
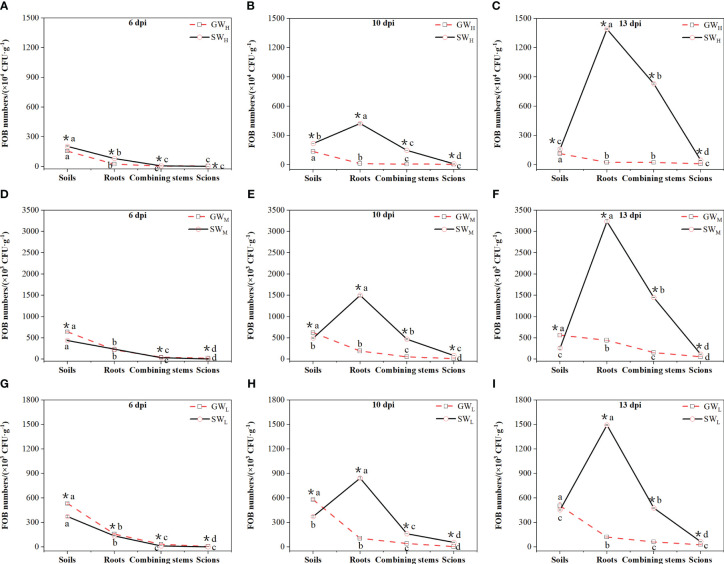
**(A–I)** Differences in the number of *Fusarium oxysporum* in soil, roots, combined stems, and scions. dpi, days post inoculation, GW_H_, Grafted wax gourd high concentration, GW_M_, Grafted wax gourd medium concentration, GW_L_, Grafted wax gourd low concentration, SW_H_, Self-grafted wax gourd high concentration, SW_M_, Self-grafted wax gourd medium concentration, SW_L_, Self-grafted wax gourd low concentration. FOB, *Fusarium oxysporum* Schl. f. sp. *benincasae*. The data are presented as the means ± SEs of three replicates. *indicates that different grafted plants with the same index differ significantly at the 0.05 level; lowercase letters indicate that different indices of the same grafted plant differ significantly at the 0.05 level.

At all cultivation times, the amount of FOB in the soil in the GW_H_, GW_M_, and GW_L_ treatments was greater than that in the roots, and the amount of FOB in the soil in the SW_H_, SW_M_, and SW_L_ treatments was also greater than that in the roots at 6 dpi; however, the opposite results were observed at 10 dpi and 13 dpi, when the amount of FOB in the soil was lower than that in the roots. Along with prolongation of the culture time, the amount of FOB in the plants of all treatments decreased from root to the scions gradually; for self-grafted wax gourd, the amount of FOB in the roots of SW_H_, SW_M_, and SW_L_ treatments were increased gradually, for grafted wax gourd, the amount of FOB in the roots of the GW_H_, GW_M_, and GW_L_ treatments showed no significant difference. The amount of FOB in the roots of SW was obviously higher than that in GW at the three concentrations of inoculated soil at 10 dpi (**Figure 3**).

### 3.3 Effects of pumpkin grafting on roots antimicrobial compound, physical barrier indicators, and their defense enzyme activities

Only the medium concentration treatment (8.0 × 10^5^ CFU·g^−1^) (SW_M_) was able to show the entire process of wax gourd wilt disease (**Figure 2**). Therefore, in our study, therefore, in our study, we select SW_M_ as the analytical object and GW_M_ was used as the contrast to execute the physiological analysis of root disease resistance.

#### 3.3.1 Total phenol and lignin contents

**Figure 4:**
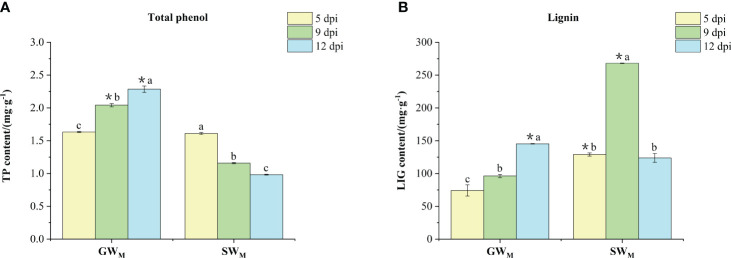
The content of total phenol **(A)** and lignin **(B)** in the roots of grafted wax gourd (GW) and self-grafted wax gourd (SW). dpi, days post inoculation, GW_M_, Grafted wax gourd medium concentration, SW_M_, Self-grafted wax gourd medium concentration. The data are presented as the means ± SEs of three replicates. *indicates that different grafted plants of the same index differ significantly at the 0.05 level. The lowercase letters indicate that different indices of the same grafted plant differ significantly at the 0.05 level.

With prolongation of the inoculation time, the contents of TP and LIG in the GW_M_ treatments significantly increased, the content of TP in the SW_M_ treatments significantly decreased, and the content of LIG first increased and then decreased (**Figures 4A, B**). At 5 dpi, TP content in the GW_M_ treatment was not significantly different from that in the SW_M_ treatment, whereas the LIG content was significantly lower than that in the SW_M_ treatment. At 9 dpi, the TP content in the GW_M_ treatment was significantly greater than that in the SW_M_ treatment, whereas the content of LIG was significantly lower than that in the SW_M_ treatment. At 12 dpi, the TP and LIG contents in the GW_M_ treatment group were significantly greater than those in the SW_M_ treatment group (**Figures 4A, B**).

#### 3.3.2 Activities of phenylalanine ammonia lyase and polyphenol oxidase

**Figure 5:**
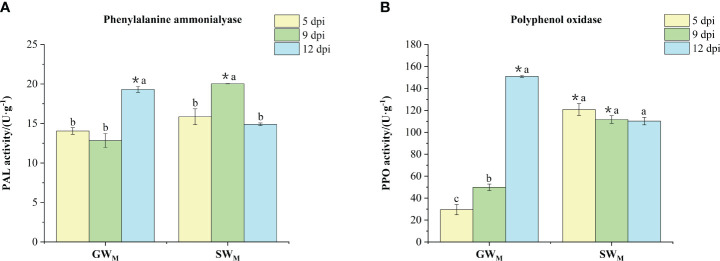
The activities of phenylalanine amino lyase **(A)** and polyphenol oxidase **(B)** in the roots of grafted wax gourd (GW) and self-grafted wax gourd (SW). dpi, days post inoculation, GW_M_, Grafted wax gourd medium concentration, SW_M_, Self-grafted wax gourd medium concentration. The data are presented as the means ± SEs of three replicates. *indicates that different grafted plants of the same index differ significantly at the 0.05 level. The lowercase letters indicate that different indices of the same grafted plant differ significantly at the 0.05 level.

Along with prolongation of the inoculation time, PAL activity in the GW_M_ treatments decreased first and then increased significantly, while PPO activity increased significantly. PAL activity in the SW_M_ treatment increased first and then decreased, while PPO activity did not significantly differ among the three culture times (**Figures 5A, B**). At 5 dpi, there was no significant difference in PAL activity between the SW_M_ and GW_M_ treatments, whereas the PPO activity in the GW_M_ treatment was significantly lower than that in the SW_M_ treatment. At 9 dpi, PAL and PPO activities in the GW_M_ treatment group was significantly lower than that in the SW_M_ treatment group. At 12 dpi, PAL and PPO activities in the GW_M_ treatment group were significantly greater than those in the SW_M_ treatment group (**Figures 5A, B**).

#### 3.3.3 Peroxidase activity

**Figure 6:**
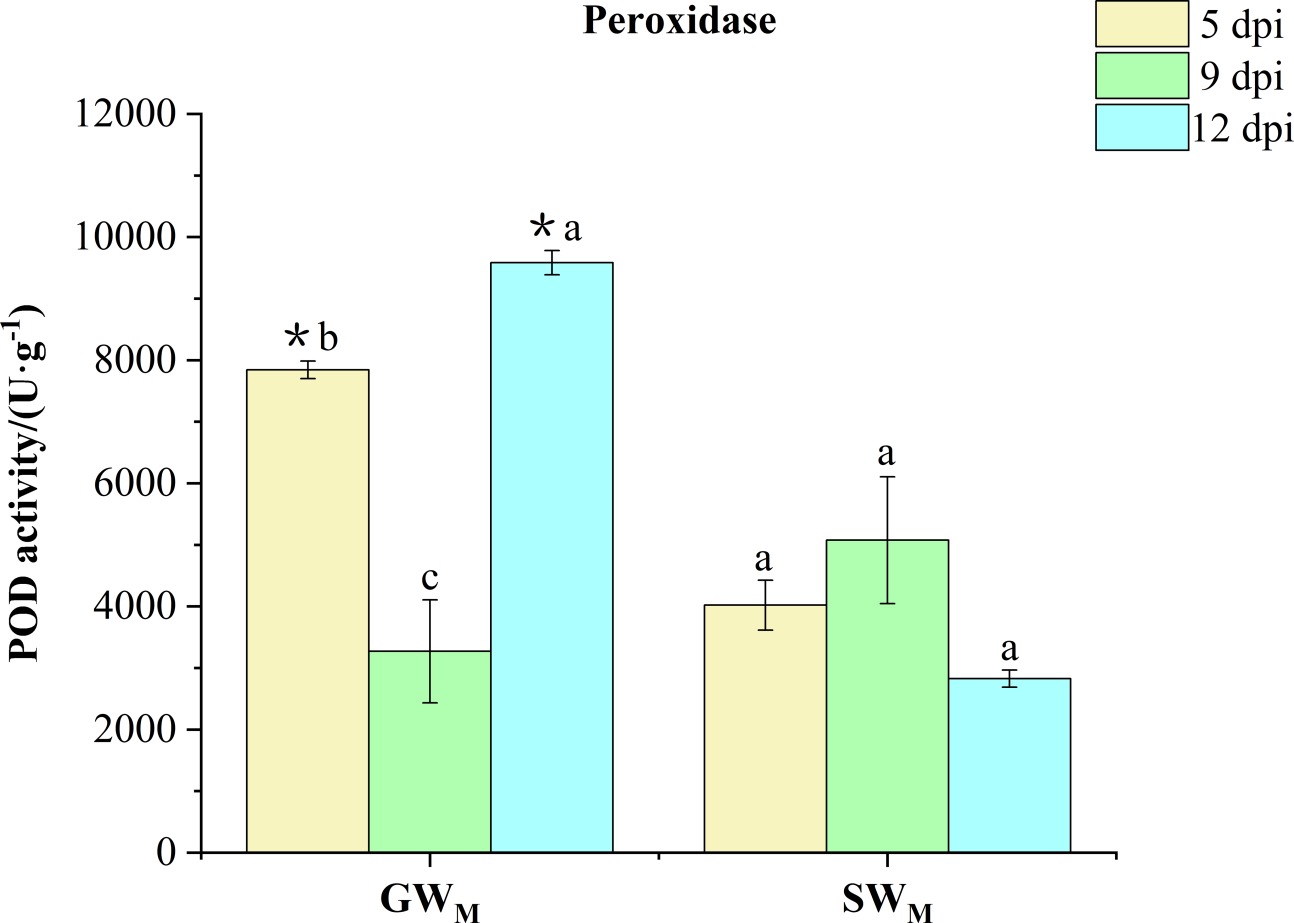
Activity of peroxidase in response to grafted wax gourd (GW) and self-grafted wax gourd (SW) in roots. dpi, days post inoculation, GW_M_, Grafted wax gourd medium concentration, SW_M_, Self-grafted wax gourd medium concentration. The data are presented as the means ± SEs of three replicates. *indicates that different grafted plants of the same index differ significantly at the 0.05 level. The lowercase letters indicate that different indices of the same grafted plant differ significantly at the 0.05 level.

Along with prolongation of the inoculation time, POD activity in the GW_M_ treatment first decreased and then increased, whereas there was no significant difference in POD activity in the SW_M_ treatment. At 5 dpi and 12 dpi, POD activity in the GW_M_ treatments was significantly greater than that in the SW_M_ treatments. At 9 dpi, there was no significant difference in POD activity between GW_M_ and SW_M_ treatments (**Figure 6**).

#### 3.3.4 Chitinase activity

**Figure 7:**
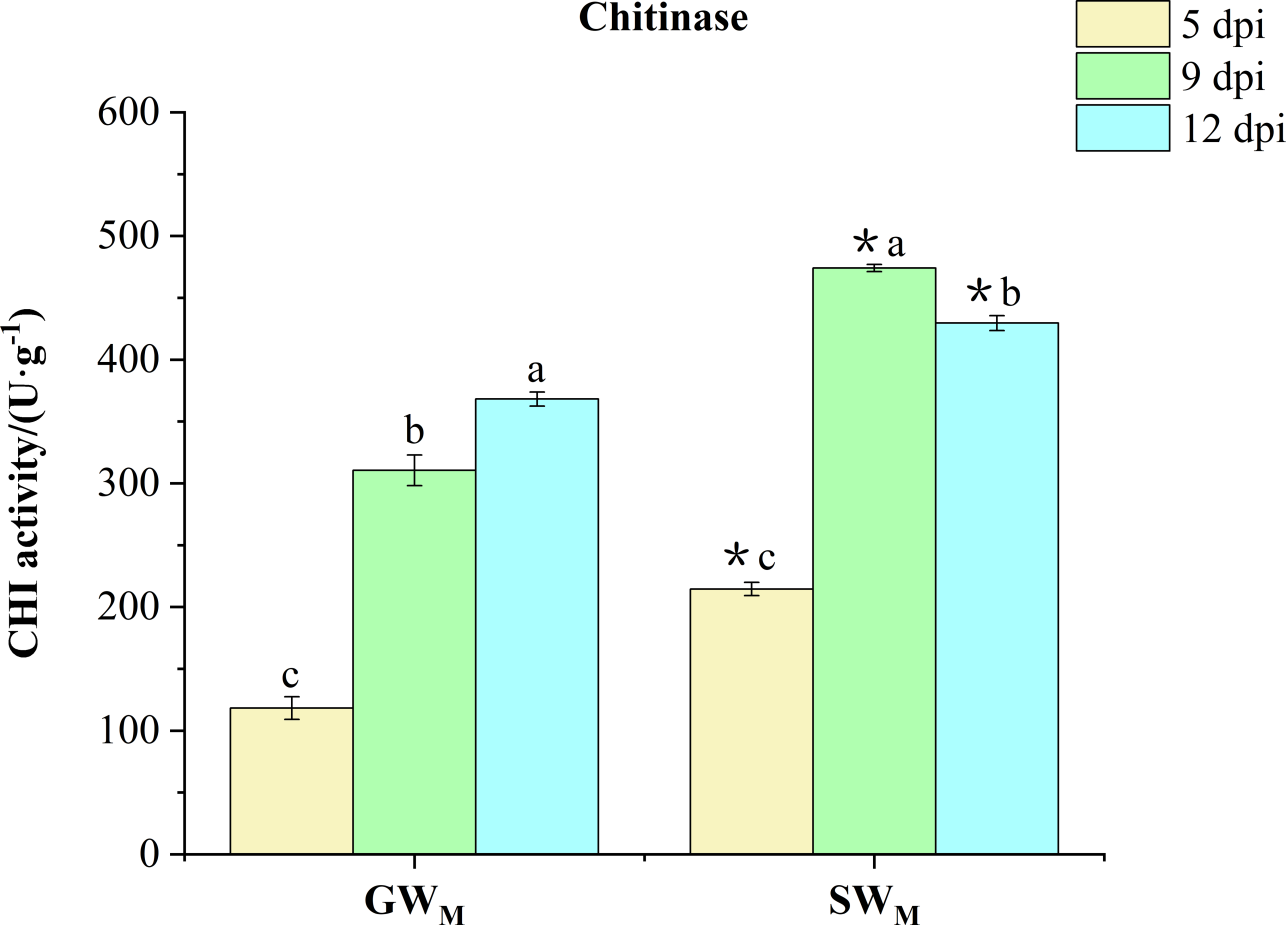
Chitinase activity in response to grafted wax gourd (GW) and self-grafted wax gourd (SW) in roots. dpi, days post inoculation, GW_M_, Grafted wax gourd medium concentration, SW_M_, Self-grafted wax gourd medium concentration. The data are presented as the means ± SEs of three replicates. *indicates that different grafted plants of the same index differ significantly at the 0.05 level. The lowercase letters indicate that different indices of the same grafted plant differ significantly at the 0.05 level.

Along with prolongation of the inoculation time, CHI activity in the GW_M_ treatment increased significantly, whereas that in the SW_M_ treatment increased first and then decreased. At all culture times, the CHI activity in the SW_M_ treatment was significantly greater than that in the GW_M_ treatment (**Figure 7**).

#### 3.3.5 Correlation analysis

**Figure 8:**
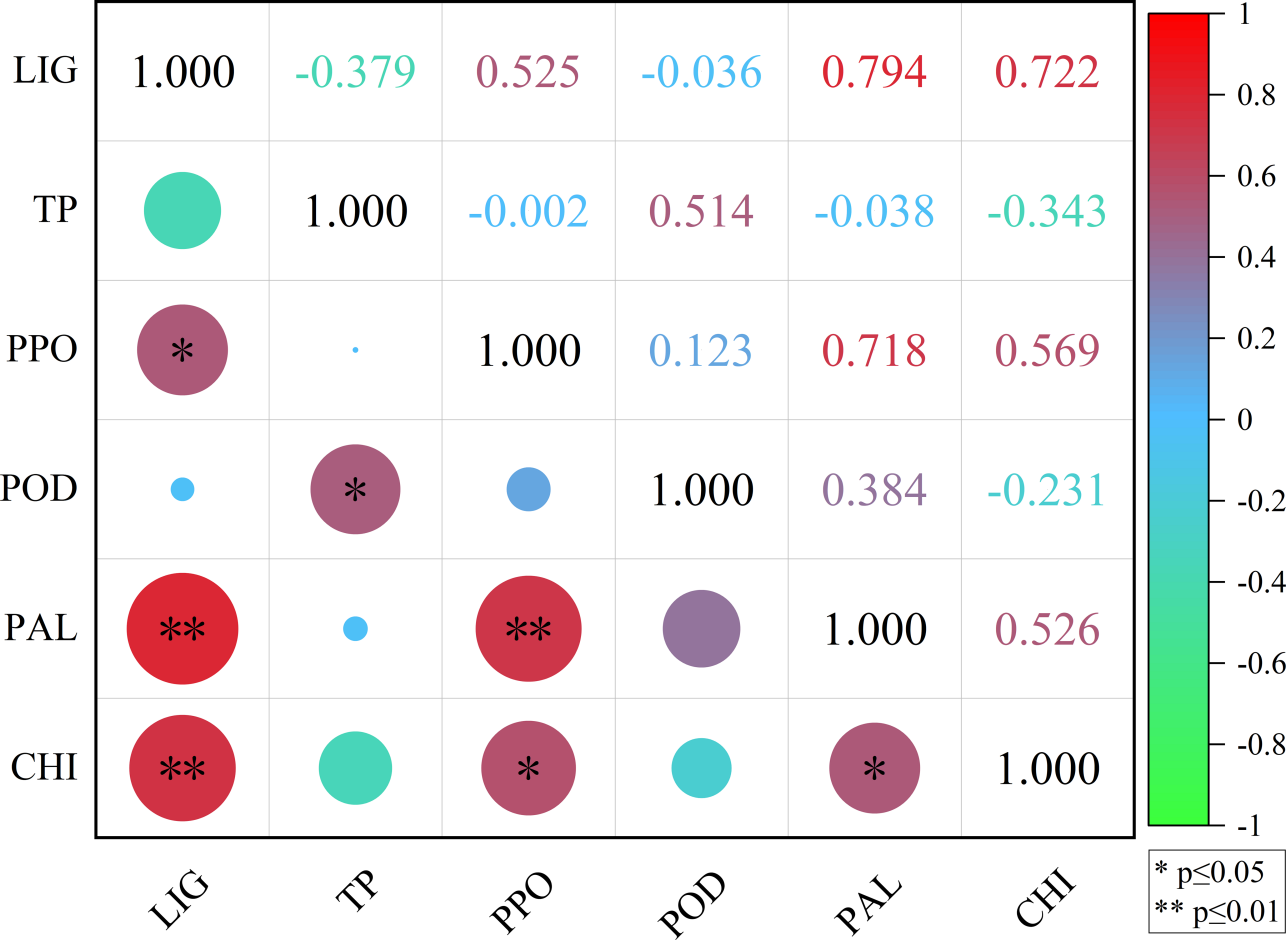
Correlation analysis between LIG, TP, PPO, POD, PAL, and CHI. LIG, lignin; TP, total phenol; PPO, polyphenol oxidase; POD, peroxidase; PAL, phenylalanine ammonia lyase; CHI, chitinase.

The correlation coefficients of LIG with PPO, TP with POD, PPO with CHI, and PAL with CHI were 0.525, 0.514, 0.569, and 0.526, respectively (**Figure 8**), indicating a significant positive correlation (p<0.05). The correlation coefficients of LIG with PAL, LIG with CHI, and PPO with PAL were 0.794, 0.722, and 0.718, respectively (**Figure 8**), indicating an extremely significant positive correlation (p<0.01).

## 4 Discussion

Grafting has many advantages (Awu et al., 2023) in improving plant growth characteristics, increasing yield and quality, and enhancing plant stress resistance. The characteristics of rootstock types affect the resistance and tolerance of grafted plants to soil-borne diseases, as the substances in the scion and rootstock of grafted plants are constantly exchanged (Pina and Errea, 2005; Bletsos and Olympios, 2008; Aloni et al., 2010). Therefore, tolerant rootstock grafting can effectively prevent and control soil-borne diseases. The occurrence of soil-borne diseases can be determined by directly observing the disease symptoms of the grafted plants. To realize an early prediction and evaluate the disease resistance effect of the stock, we can also measure the amount of FOB in the grafted plants. In this study, through a pot simulation test, it was found that when used as rootstock, “Haizhan 1” pumpkin, did not show any symptoms of disease, whereas “Tiezhu 2” wax gourd did infect with wilt which was consistent with the results of Yuan et al. (2021) and further indicated that “Haizhan 1” pumpkin was highly resistant to *Fusarium* wilt (**Figure 2**). This study also revealed that the number of FOB cells in SW roots increased when the culture time was prolonged. There was no significant difference in the number of FOB in GW roots with increasing culture time. Regardless of the concentration of soil that was cultured up to 10 dpi, the number of FOB in the SW roots was significantly greater than that in the GW roots, and the difference became more obvious when culture time was prolonged. These results indicated that, compared with “Tiezhu 2” rootstock, “Haizhan 1” and pumpkin rootstock could effectively prevent the proliferation of FOB at the root, and subsequently effectively inhibit the occurrence of wax gourd wilt.

Plants under adverse or disease conditions often exhibit two defense mechanisms: one is through the inherent physical barrier structures and antimicrobial compounds with certain disease resistance functions, and the other is through the protective enzyme system of the plants to protect themselves from adverse environmental influences, thereby exhibiting the quality of disease resistance (Zheng et al., 2017; Pan et al., 2020; He, 2022). Physical barriers function as a defense method to inhibit the occurrence of wax gourd wilt (Zheng et al., 2017; Swaminathan et al., 2022). For example, cutin, wax, cork, and lignin structures of the cell wall can effectively resist the invasion and spread of pathogenic fungi. LIG is a cross-linked molecule that is polymerized by many phenylpropane monomers and deposited in the cell wall of plant lignified tissues to become a barrier to prevent the growth and reproduction of pathogenic fungi (Tronchet et al., 2010). In this study, the content of LIG in GW_M_ roots was significantly lower than that in SW_M_ roots at 5 dpi and 9 dpi but was significantly greater than that in SW_M_ roots at 12 dpi. Moreover, the LIG and TP content in the GW_M_ roots continued to increase (**Figure 4**). Hussain et al. (2023) reported that pigeon pea activates lignin expression in the lignin synthesis pathway to increase the resistance of plants to *Fusarium* wilt. LIG blocks cell walls, forms a physical barrier, reduces the spread of fungal mycelial toxins, and reduces the amount of nutrients transported by host cells to pathogenic fungi (Siddaiah et al., 2017). *Fusarium* wilt symptoms developed earlier in SW; therefore, more LIG was formed in the roots of SW plants to prevent FOB invasion at 9 dpi. However, at 12 dpi, *Fusarium* wilt was so severe in SW plants that there was no longer enough LIG to be synthesized; therefore, the LIG content in the roots of SW was reduced. Although GW did not cause any symptoms of *Fusarium* wilt, the roots of GW synthesized more LIG over a prolonged period to resist FOB invasion. The precursor phenolic substances of LIG biosynthesis are oxidized to quinone, and lignin is synthesized and accumulates in the cell wall (Liu et al., 2009). Taken together, these findings demonstrate that when FOB invasion occurs, the roots of the “Haizhan 1” pumpkin rootstock can continuously synthesize LIG to form a physical barrier that resists FOB infection. The wilt-resistant antimicrobial compounds in plants are secondary metabolites. For example, phenylpropanoid metabolism is an important metabolic pathway for the generation of secondary metabolites such as phytoalexin, lignin, and phenolic compounds, which contribute to disease resistance. Phenolic acids are secondary metabolites with high disease resistance in plants. Most phenolic acids are antibiotics that kill infectious pathogens (Schnur et al., 2021). Therefore, phenolic acids can improve plant defense functions. At 5 dpi, the content in the GW_M_ treatment was not significantly different from that in the SW_M_ treatment. At 9 dpi and 12 dpi, the TP content in the roots of the plants in the GW_M_ treatment was significantly greater than that in the SW_M_ treatment. Moreover, with the extension of culture time, the TP content in the roots of plants in the GW_M_ treatment group continued to increase (**Figure 4**). In summary, the roots of the “Haizhan 1” pumpkin rootstock continued to synthesize disease-resistant phenolic substances, kill pathogenic fungi, and resist FOB infection through the chemical substances of the plants themselves.

Although the “Haizhan 1” pumpkin rootstock can effectively prevent the proliferation of FOB in roots, however, FOB was still detected in the roots of grafted wax gourd, but no infection of the stem led to wax guard wilt, which indicated that changes in the protective enzyme system in the plant may have played an important role in preventing the development of wilt in grafted wax gourd For example, PAL is a key enzyme in the shikimic acid pathway (Dong and Lin, 2021), that can form disease-resistant secondary substances, including LIG and phenolic compounds. PPO can directly use O_2_ as an oxidation substrate to oxidize phenol to quinone, which kills and inhibits the growth and reproduction of pathogenic fungi (Raj et al., 2006). PODs are present in the cell wall, participate in the formation of LIG, effectively remove reactive oxygen species, and oxidize phenolic substances to quinones, which have toxic effects on pathogenic fungi (Jiang et al., 2014). Chitin is one of the main components of the cell walls of many pathogenic microbes (especially fungi) that harm plants. CHI is an enzyme that hydrolyzes chitin, and CHI inhibits pathogenic fungi by degrading the cell wall (Meena et al., 2017). In the present study, as the culture time increased, the activities of PAL and POD enzymes in the GW_M_ roots first decreased and then increased, whereas the activities of the PPO and CHI enzymes continued to increase. The activities of PAL, POD, and CHI enzymes in the SW_M_ roots first increased and then decreased, while the activities of PPO enzymes continued to decrease (**Figures 5**-**7**). Sadeghpour et al. (2022) reported that after muskmelon was infected by *F. oxysporum*, PPO, POD, and CHI enzyme activities were upregulated in disease-resistant varieties compared with susceptible varieties, suggesting that these enzymes play an active role in resistance to *F. oxysporum*. Xu et al. (2021) also reported that *Chi2* and *Chi14* genes in cucumber were significantly upregulated after infection with *F. oxysporum*, which significantly inhibited the growth of *F. oxysporum* and promoted disease resistance. These results indicated that, compared with the “Tiezhu 2” wax gourd rootstock, the “Haizhan 1” pumpkin rootstock inhibited the proliferation of *F. oxysporum* by improving the activities of root defense enzymes (PAL, PPO, POD, and CHI) and enhancing the resistance of plants to *Fusarium* wilt when infected by FOB. For SW_M_-treated plants infected with wax gourd wilt disease at 6 dpi (**Figure 2**), PAL, PPO, POD, and CHI enzymes were activated, and the synthesis of LIG and its phenolic substances accelerated (Lv et al., 2020; Yu, 2022). However, when wax gourd wilt disease became more severe at 13 dpi (**Figure 2**), the activities of PAL, PPO, POD, and CHI responded with a significant decrease in the synthesis of LIG and its phenolic substances.

Finally, the correlation analysis of each index of disease-resistance-related enzymes can determine the correlation between them. The results showed significant or extremely significant positive correlations between LIG and PAL, PPO and CHI, TP, and POD (**Figure 8**). Wang et al. (2017) found that the increase of PAL activity coincided with the increase of LIG content when plants were infected by fungi. Liu et al. (2016) found that POD enzyme activity and LIG content in vegetative organs of melonis is increased after inoculation with *Fusarium* wilt, indicating that LIG and enzymes related to LIG synthesis played an important role in the resistance to *Fusarium* wilt. Jia and Xu (2016) found that PPO activity of disease-resistant varieties increased rapidly and LIG content gradually after inoculation of root-knot nematodes with different resistances; the increase on disease-resistant varieties was significantly higher than that of highly susceptible varieties, indicating that disease-resistant varieties deposited more LIG to participate in the construction of the defense system and enhance the physical barrier against pathogen infection, thus slowing the infection of root-knot nematodes. Singh (2023) studied the relationship between phenols and antioxidant enzymes in spinach leaves treated with different concentrations of salicylic acid and found that the content of phenols (TP) was positively correlated with POD activity, helping to protect plants from environmental stress. These studies revealed that the enhanced activities of POD, PAL, and PPO were conducive to the synthesis of more phenolic substances (TP) and LIG, killing FOB, strengthening the physical barrier, and resisting FOB infection.

## 5 Conclusion

In conclusion, the “Haizhan 1” pumpkin rootstock can effectively prevent the proliferation of FOB in roots and subsequently inhibit the occurrence of wax gourd wilt. When FOB infects roots, a self-defense mechanism begins, the activity of disease-resistant enzymes (e.g., PPO, POD, PAL, and CHI) increases, secondary metabolism accelerates, and additional LIGs and phenolic substances (TPs) are generated. These results support the high resistance of grafted wax gourd to wax gourd wilt disease.

## Data availability statement

The original contributions presented in the study are included in the article/supplementary material. Further inquiries can be directed to the corresponding authors.

## Author contributions

HF: Methodology, Writing – original draft, Data curation. JF: Data curation, Methodology, Writing – original draft. BZ: Funding acquisition, Writing – review & editing. HW: Data curation, Writing – original draft. DL: Funding acquisition, Writing – review & editing. ZL: Funding acquisition, Writing – review & editing, Conceptualization.
